# Phytochemical Analysis and Anti-Ulcer Potential of Phenolic Compounds of *Inonotus nidus-pici* Pilát

**DOI:** 10.3390/ph18091265

**Published:** 2025-08-25

**Authors:** Iliya Slavov, Nadezhda Ivanova, Maya Radeva-Ilieva, Stanila Stoeva-Grigorova, Deyan Dzhenkov, Kaloyan D. Georgiev

**Affiliations:** 1 Department of Biology, Faculty of Pharmacy, Medical University of Varna, 9000 Varna, Bulgaria; ijelev80@abv.bg; 2 Department of Pharmaceutical Technologies, Faculty of Pharmacy, Medical University of Varna, 9000 Varna, Bulgaria; nadejda.ivanova@mu-varna.bg; 3 Department of Pharmacology, Toxicology and Pharmacotherapy, Faculty of Pharmacy, Medical University of Varna, 9000 Varna, Bulgaria; maya.radeva@mu-varna.bg (M.R.-I.); stoeva.st@mu-varna.bg (S.S.-G.); 4 Department of General and Clinical Pathology, Forensic Medicine and Deontology, Division of General and Clinical Pathology, Faculty of Medicine, Medical University of Varna, 9000 Varna, Bulgaria; ddzhenkov@gmail.com

**Keywords:** *Inonotus nidus-pici*, phenols, HPLC, antioxidants, anti-ulcer activity

## Abstract

**Background/Objectives**: Fungotherapy has long been recognized as a therapeutic approach for treating and preventing various diseases. As an important representative of the so-called functional mushrooms, Chaga plays a crucial role in this system. Since this species is of limited distribution in Bulgaria, we are interested in studying a related but different species, *Inonotus nidus-pici* Pilát, with potential benefits for human health. **Methods**: The phytochemical composition of phenolic compounds in the studied species was analyzed using spectrophotometric methods and high-performance liquid chromatography (HPLC). Additionally, antioxidant activity was assessed using various assays, and the gastroprotective effect was evaluated in experimental rat models with indomethacin-induced gastric damage. **Results**: The quantities of the main classes of phenolic compounds in the studied object were determined, and an enriched phenolic extract (EPE) was obtained. The amount of phenolic compounds, in decreasing order, is as follows: tannins (1.67 ± 0.02%), phenolic acids (1.50 ± 0.09%), and flavonoids (1.24 ± 0.04%). Quercetin was the most present flavonoid (15.95 ± 0.05 mg/g DWE), followed by (+)-catechin (9.86 ± 0.15 mg/g DWE) and kaempferol (1.67 ± 0.09 mg/g DWE) in the enriched phenolic extract. The quantity of other established compounds was significantly lower. Of all ten phenolic acids identified in the same extract, the highest concentration was found only for rosmarinic acid (6.41 ± 0.08 mg/g DWE) and somewhat for *p*-coumaric acid (2.13 ± 0.12 mg/g DWE). Among all the applied methods regarding antioxidant activity, the highest potential of the extract for reducing copper ions was the most pronounced (1506.93 μM TE/g DWE), and the ability of the extract to reduce iron ions was almost the same (1354.05 μM TE/g DWE). In the experimental indomethacin-induced gastric ulcer rat model, EPE (25 mg/kg and 10 mg/kg) demonstrated a dose-dependent gastroprotective effect. **Conclusions**: The results of the experiments confirm the potential of the wood fungus species as a source of valuable biologically active compounds with beneficial and pharmacological effects. However, further studies are needed to fully determine its chemical composition and the biological activities related to it.

## 1. Introduction

Since ancient times, wood mushrooms have served both as a food source and as remedies for conditions such as cancer, inflammation, and immune system disorders—especially within traditional Far Eastern medicine [1]. In Europe, one of the earliest records of this use has been found in Hippocrates’ work, Corpus Hippocraticum. Known as the father of medicine, Hippocrates used infusions made from mushrooms externally to cleanse wounds [2]. Currently, modern fungotherapy is a promising system for the treatment and prevention of diseases using functionally active medicinal fungi [3].

A prominent representative of the group of medicinal mushrooms in Europe is *Inonotus obliquus*, well-known as “Chaga”. *I. obliquus* is highly valued in traditional medicine, especially in Russia, Poland, and northern countries, and is currently one of the most popular medicinal mushrooms with antitumor, antioxidant, antimicrobial, antiviral, and immunomodulating properties due to its wide range of valuable, structurally diverse, and biologically active natural compounds such as polysaccharides, triterpenoids, phenol derivatives, and many others [4].

*Inonotus nidus-pici* Pilát is another member of the genus *Inonotus*, previously considered a form of the Chaga, but it has been proven to be a related species. It is a wood-destroying mushroom widespread in Southeastern European countries. The hosts of this fungus species are different oaks, with *Quercus cerris* (L.) being the primary host [1]. The sclerotia of this species are perennial, with the annual formation of new tissue in the periphery of old ones. They are initially soft and yellowish-brown, turning dark brown to black later, and are usually formed around injured tissue on the surface of trunks. The external symptoms of injuries are expressed on the stem, most commonly holes of woodpeckers, with a peripherally dark-colored bark with a shaped bump. On the other hand, internal injuries are well-expressed through brown core rot, extending from the site of infection. The wood rotates in width and height and does not affect the white core [1,5].

Unlike Chaga, there are almost no data in the literature on phytochemical compounds in *I. nidus-pici* or its medicinal properties. The only one studied so far is on the methanol extract of the species, and the following compounds have been identified: citropremid, 3,4-dihydroxybenzalacetone, lanosterol, ergost-6,8,22-trien-3β-ol, and ergosterol peroxide. The compounds have also been tested for their antioxidant and antimicrobial properties against several bacterial and fungal strains, as well as their antiproliferative activity on three human cancer cell lines (MES-SA, MES-SA/Dx5, and A431) [1].

Therefore, we aimed to investigate the phytochemical composition and potential biological effects (i.e., anti-ulcer and antioxidant activities) of the enriched phenolic extracts from this species.

## 2. Results

The analysis of the phenolic fraction began with the study of its phytochemical composition and the identification of some important phenolic compounds. Furthermore, the phenolic-enriched fraction demonstrated antioxidant effects as well as anti-ulcer potential in experimental animals.

### 2.1. Spectrophotometric Analysis of Main Phenolic Compounds

**Figure 1:**
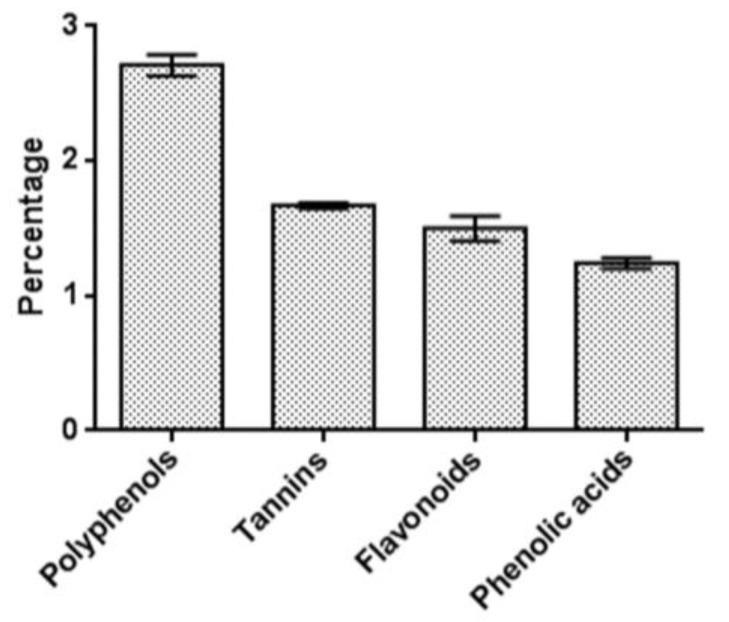
Quantity of main phenolic compounds in *I. nidus-pici* sclerotia (% dry-weight mass of substances).

Initially, the amount of main phenolic compounds in the mushroom-tested sample was measured to focus our attention on the relevant groups of biologically active substances with presumed value. The amount of water-soluble phenolic compounds and tannins was expressed as pyrogallol equivalent (in % dry-weight mass of substances). The *I. nidus-pici* from Bulgaria contained 2.71 ± 0.08% of total water-soluble polyphenols, with 1.67 ± 0.02% identified as tannins and almost the same amount of the established phenolic acids (1.50 ± 0.09%). Compared to other groups, flavonoids were present in the lowest quantity (1.24 ± 0.04%) (Figure 1). All the spectrophotometric methods used in this study further outlined the need for an in-depth analysis using modern instrumental methods.

### 2.2. HPLC Analysis of Flavonoid and Phenolic Acid Profiles in EPE (Enriched Phenolic Extract) from the Dry-Weight Extract (DWE)

**Figure 2:**
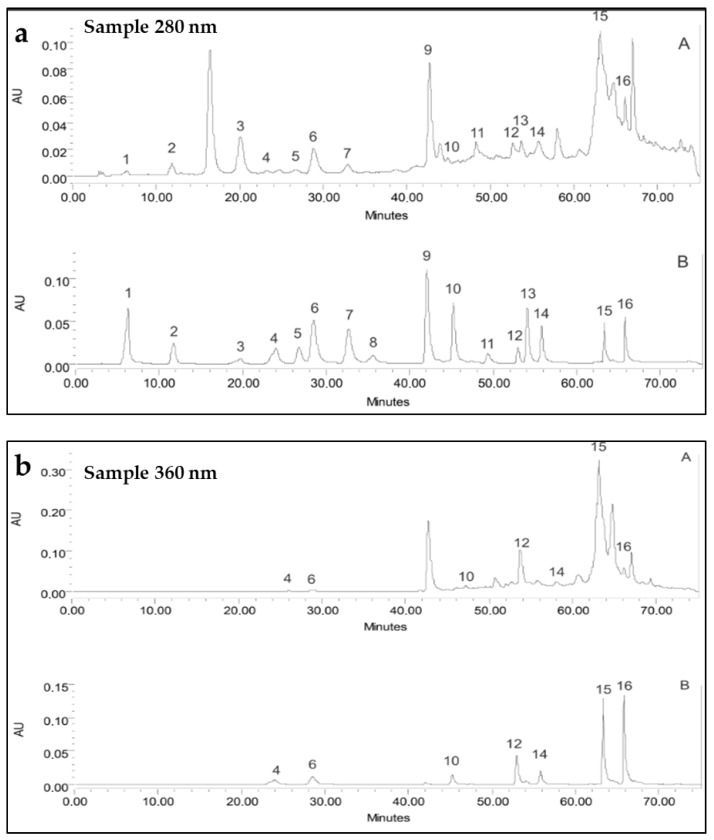
HPLC chromatograms of phenolic compounds from *I. nidus-pici* (A) and used standards (B) determined at 280 nm (**a**) and 360 nm (**b**). (**a**) Peaks (280 nm): 1—Gallic acid, 2—Protocatechuic acid, 3—(+)-Catechin, 4—Chlorogenic acid, 5—Vanillic acid, 6—Caffeic acid, 7—Syringic acid, 8—(−)-Epicatechin, 9—*p*-Coumaric acid, 10—Ferulic acid, 11—Salicylic acid, 12—Rutin, 13—Hesperidin, 14—Rosmarinic acid, 15—Quercetin, and 16—Kaempferol. (**b**) Peaks (360 nm): 4—Chlorogenic acid, 6—Caffeic acid, 10—Ferulic acid, 12—Rutin, 14—Rosmarinic acid, 15—Quercetin, and 16—Kaempherol.

Quercetin was better represented as aglycone (15.95 ± 0.05 mg/g DWE), followed by kaempferol (1.67 ± 0.09 mg/g DWE). At the same time, the quantity of the glycosides hesperidin and rutin was significantly lower (0.83 ± 0.03 mg/g DWE and 0.44 ± 0.14 mg/g DWE, respectively). Of the other detected flavonoid compounds, the flavan-3-ol (+)-catechin occurred in higher quantity (9.86 ± 0.15 mg/g DWE), while (−)-epicatechin was not identified in the present analysis. Ten phenolic acids were identified in the enriched phenolic extract (EPE) from the investigated fungus. The highest concentration was established only for rosmarinic acid (6.41 ± 0.08 mg/g DWE) and somewhat for *p*-coumaric acid (2.13 ± 0.12 mg/g DWE), followed by ferulic and salicylic acids. Chlorogenic, protocatechuic, caffeic, vanillic, syringic, and gallic acids were also present (Table 1 and Figure 2).

### 2.3. Antioxidant Capacity of EPE

Four in vitro assays (Table 2) were conducted to investigate the antioxidant capacity of the EPE used. All applied methods reported positive results, but the extract’s potential to reduce copper ions was the most pronounced (1506.93 mM TE/g DWE), and the extract’s ability to reduce iron ions was almost the same (1354.05 mM TE/g DWE). Regarding the radical scavenging activity, the difference was about 25 times greater in favor of ABTS^•+^ compared to DPPH^•+^.

The established high content of some individual phenols may explain the demonstrated powerful antioxidant effect of the extract.

### 2.4. Macroscopic Evaluation of Gastric Ulcers

No gastric ulcers were observed in the control group, while in the Indomethacin group, extensive gastric ulcers were detected. The macroscopic evaluation shows that phenols (in the investigated EPE), isolated from *Inonotus nidus-pici*, have a significant dose-dependent anti-ulcer effect in rats, similar to that of famotidine.

The ulcer index (UI) for all groups and the percentage of protection (PP) for pretreated groups (III, IV, and V) were calculated to assess the severity of mucosal lesions. The results are shown in Table 3:

### 2.5. Histological Analysis of Gastric Ulcers

**Figure 3:**
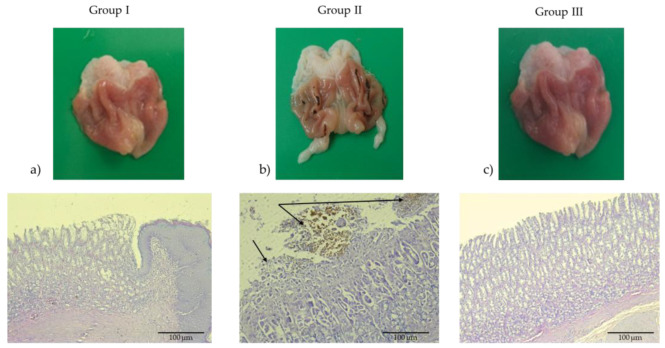
Macroscopic and photomicrographs of stomachs with H&E (200× magnification, scale bar 100 μm). (**a**) The control group received 1 mL of saline orally for 14 consecutive days. (**b**) *Indomethacin*-treated group (induction dose: 40 mg/kg); erosions (arrow left) and erosions with haemosiderin (arrow right). (**c**) Group III pretreated with famotidine (20 mg/kg) for 14 consecutive days prior to ulcer induction. The group showed no damage to the gastric mucosa, both macroscopically and with histological analysis.

Histopathological examination of hematoxylin–eosin-stained longitudinal sections from the control group (Group I) revealed normal gastric histoarchitecture, with intact surface epithelium, well-preserved foveolar and glandular zones, and no evidence of inflammatory infiltrates or hemorrhage. Conversely, gastric sections from the indomethacin-treated group (Group II) demonstrated significant mucosal injury, including widespread epithelial cell degeneration, surface erosions, and focal areas of hemorrhage (deposits of hemosiderin), which are indicative of acute inflammation (Figure 3). In the group pretreated with famotidine (20 mg/kg) for 14 consecutive days prior to ulcer induction, the gastric mucosa appeared to be preserved, both macroscopically and microscopically, with the maintenance of epithelial integrity and the absence of inflammatory or hemorrhagic changes.

**Figure 4:**
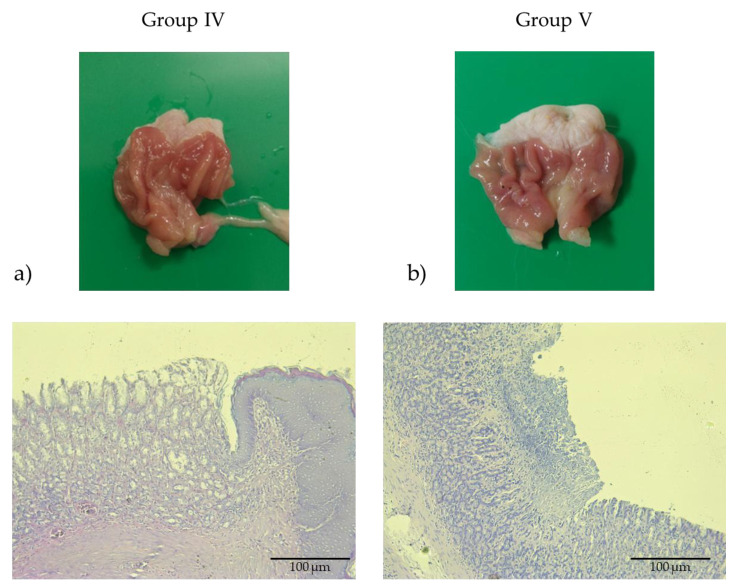
Macroscopic images and photomicrographs of stomachs with H&E (200× magnification, scale bar 100 μm). (**a**) Group IV pretreated with EPE from *Inonotus nidus-pici* (25 mg/kg) for 14 consecutive days prior to ulcer induction. (**b**) Group V pretreated with EPE from *Inonotus nidus-pici* (10 mg/kg) for 14 consecutive days prior to ulcer induction. The groups showed dose-dependent protective effects, both macroscopically and with histological analysis.

Groups IV and V, pretreated with catechins isolated from *Inonotus nidus-pici* at doses of 25 mg/kg and 10 mg/kg, respectively (Figure 4a,b), exhibited a dose-dependent gastroprotective response against indomethacin-induced mucosal injury. At the 25 mg/kg dose, phenols from EPE markedly attenuated indomethacin-associated histopathological alterations, with mucosal preservation comparable to that observed in the famotidine-treated group.

## 3. Discussion

Most current studies on the species *Inonotus nidus-pici* were done for the first time. For the first time, flavonoids and phenolic acids have been studied and quantified in this mushroom. Garádi et al. (2021) [1] established several other compounds with different structures in a previous work, but it did not include flavonoids and phenolic acids. All this identifies the species as a promising source of phenolic active substances.

Data on phenolic components are available for a related and widely studied species, *I. obliquus* (Chaga). The variety of phenols from these two groups has been pointed out in numerous works. Some of the valuable established flavonoids are kaempferol, quercetin, catechin 7-xyloside, tangeretin, isorhamnetin-3-*O*-glucoside, astilbin [6], myricetin, naringenin, isorhamnetin [7], catechin [8], epicatechin-3-gallate, epigallocatechin-3-gallate, naringin [9], rutin [10], and hispidine [11]. Among the established representatives of phenolic acids are hydroxycinnamic derivatives and derivatives of benzoic acid: *p*-coumaric acid [6], caffeic acid [6,7], syringic acid [7,8], ferulic acid [7,9], salicylic acid [7], protocatechuic acid [4,7], gallic acid [6], vanillic acid [7], and ellagic acid [11]. In addition, a full phenolic description was presented by Ern et al. (2024), along with other flavonoid and phenolic acid derivatives in Chaga mushroom [11]. In addition to these, an abundance of different secondary metabolites were reported, including terpenoids, sterols, sesquiterpenes, melanins, polysaccharides, and others [4].

In the only study, to date, by Garádi et al. (2021), an antioxidant effect for *I. nidus-pici* was observed for the compound 3,4-dihydroxybenzalacetone using the DPPH^•+^ method, with a value (29.7 ± 1.3 µM) similar to ours [1]. It shows the necessity of using different techniques for quantifying the antioxidant capacity of extracts in addition to their biological effects. In our research, we reported that the EPE reduces copper and iron ions more potently compared to other methods (DPPH and ABTS).

As pointed out above, flavonoids, phenolic acids, and many other phenols were primary contributors to the antioxidant potential of *I. obliquus* extracts too [11]. These mechanisms mainly relate to the ability to capture free radicals, protection against DNA damage, binding affinity to the enzyme superoxide dismutase 1, and even anticancer effects related to radical oxidation [6,7,9,11].

A low antioxidant capacity was mentioned for another *Inonotus* species, i.e., *I. hipidus*, according to the DPPH method [12]. Similar to our results, the authors used a methanol extract for this experiment. The DPPH and ABTS methods were also reviewed in another study, where the concentrations of a variety of antioxidants in ethylacetate, ethyl ether, and even water hydrodistillation extracts were described [13].

The reported antiulcerogenic effects are the first to be measured with an EPE extract from the mushroom in our study, in which methanol was used as the initial extractant. The anti-ulcer activity of *I. nidus-pici* was confirmed in our research; similarly, Xin et al. (2019) [14] established the anti-ulcer activity of the ethanol extract of *I. obliquus* using rats with induced gastric ulcers (caused by ethanol intake). As a result, the oral administration of ethanol extract showed anti-ulcer activity in all models used, probably due to the presence of various biologically active compounds. In our case, we suppose that the identified phenolic components in the EPE are responsible for that.

Ern et al. (2024), in their review article, discussed the effects of the closest fungi relative of the genus Chaga on gastrointestinal problems through various applications [11]. As the authors mention, Chaga has been used in folk medicine since the 16th century to treat gastrointestinal cancer and various stomach disorders. In their study, Szychowski et al. (2021) pointed out the ethnomedical application of Chaga, which has antiparasitic, anti-inflammatory, and gastrointestinal properties [2].

Despite the lack of clear information about the exact mechanism by which various Chaga extracts act on gastrointestinal diseases, and after careful analysis of the available scientific information in the article of Ern et al. (2024), we can infer that this is likely due to some of the following mechanisms [11]:

- Anti-inflammatory effect associated with a reduction in the expression of inflammatory cytokines (TNF-α, IL-6, and IL-1β) involved in gastrointestinal inflammation. Similar patterns have been observed in colitis, suggesting a role in alleviating conditions such as inflammatory bowel disease (IBD).
- Antioxidant properties due to complexes of terpenoids and phenols, which show significant antioxidant effects. They help reduce oxidative stress and protect the gastrointestinal mucosa from damage.
- The anticancer properties of Chaga point to its use in treating cancers, especially colorectal cancer. Compounds such as ergosterol show similar activity, inhibiting the proliferation of colorectal cancer cells and inducing apoptosis.

Another ethnopharmacological study demonstrated the antiulcerogenic properties of the other abovementioned species of this genus—*Inonotus hispidus*. In the indigenous medicine of Xinjiang province in Northeast China, local people use it to cure stomach ulcers, indigestion, diabetes, and dyspepsia [15].

As can be seen from the examples supporting our scientific hypothesis, there is a general lack of robust experimental evidence for the potential health benefits of using *Inonotus* medicinal mushrooms in the treatment of gastrointestinal diseases. All of this is a prerequisite for the need for more research on these types of functional mushrooms in the future.

## 4. Materials and Methods

The examined sterile conks of fungi *Inonotus nidus-pici* were collected in December 2024 from living *Quercus cerris* in the city of Dulovo, district of Silistra, in north-eastern Bulgaria, and were identified, based on their morphological features, by Assoc. Prof. Iliya Slavov from the Department of Biology, Sector Pharmacognosy and Pharmaceutical Botany, Faculty of Pharmacy, Medical University of Varna, Bulgaria. The samples were air-dried at room temperature, ground to 0.5 mm, and then extracted with different solvents in the performed analysis.

All used substances in spectrophotometric and HPLC analysis, indomethacin, and famotidine were purchased from Sigma-Aldrich (Darmstadt, Germany).

### 4.1. Spectrophotometric Analysis

#### 4.1.1. Spectrophotometric Quantification of Total Phenols and Tannins

The determination of phenolic metabolites, including total polyphenols and tannins, was performed according to the European Pharmacopoeia 10 [16] (p. 310), involving the Folin–Ciocalteu reagent and pyrogallol as a standard. The analyses were carried out at 760 nm. The measurements were carried out using an Ultraspec 3300 pro UV/VIS spectrophotometer (Marlboro, NJ, USA). All determinations were performed in triplicate (n = 3).

#### 4.1.2. Spectrophotometric Quantification of Flavonoids

The content of the flavonoids was determined spectrophotometrically at 420 nm by creating a complex with AlCl_3_, according to the European Pharmacopoeia 10 [16] (p. 1607). The content of flavonoids was calculated as hyperoside. The measurements were carried out using an Ultraspec 3300 pro UV/VIS spectrophotometer (Marlboro, NJ, USA). All determinations were performed in triplicate (n = 3).

#### 4.1.3. Spectrophotometric Quantification of Phenolic Acids

The total hydroxycinnamic acid derivatives in the material were determined again according to the European Pharmacopoeia 10 [16] (p. 1602), using rosmarinic acid as a reference. The analyses were carried out at 505 nm. The measurements were carried out using an Ultraspec 3300 pro UV/VIS spectrophotometer (Marlboro, NJ, USA). All determinations were performed in triplicate (n = 3).

### 4.2. Extraction Method for the Enrichment of Phenolic Extract (EPE) from Substances

Dried and ground substances were extracted with methanol in a 1:10 (*w*/*v*) ratio and evaporated in a water bath until they were dry. Briefly, 50 mL of water was added to the dried extract, as well as 5 g of sodium chloride and phosphoric acid (pH 3.5). The mixture was placed in a separating funnel four times with 50 mL of ethyl acetate, and the combined ethyl acetate extracts were washed with water until a neutral reaction occurred. After evaporating the solvent under a vacuum, a mixture of phenolics was obtained in the quantity of 4.7% [17].

### 4.3. High-Performance Liquid Chromatography Analysis of Flavonoids and Phenolic Acids from Enriched Phenol Extract (EPE)

High-performance liquid chromatography (HPLC) analysis of phenolic acids and flavonoids was performed by using Waters 1525 Binary Pump HPLC systems (Waters, Milford, MA, USA), equipped with a Waters 2484 dual Absorbance Detector (Waters, Milford, MA, USA) and a Supelco Discovery HS C18 column (5 μm, 25 cm × 4.6 mm), operated under the control of Breeze 3.30 software. Detailed conditions of HPLC analyses have been reported previously [18]. The concentration of each compound was calculated based on the external standard method and was converted to milligram (mg) of compound per gram of dry-weight extract (DWE).

### 4.4. Antioxidant Activity

The antioxidant capacity determined from all assays is expressed in millimoles of Trolox equivalent (TE) per gram of dry-weight extract (DWE). A calibration curve was created using Trolox (6-hydroxy-2,5,7,8-tetramethylchroman-2-carboxylic acid) in methanol, with concentrations ranging from 0.05 to 0.5 mmol.

#### 4.4.1. ABTS Assay

The ability of the extracts to scavenge ABTS (2,2′-azino-bis(3-ethylbenzothiazoline-6-sulfonic acid)) radicals was evaluated following the procedure in [19]. The ABTS radical cation (ABTS^•+^) was generated by combining 7.0 mmol of ABTS with 2.45 mmol of potassium persulfate, both of which were dissolved in distilled water. The mixture was left in the dark at room temperature for 16 h. Before the assay, the ABTS^•+^ solution was diluted with methanol to reach an absorbance of 1.0–1.1 at 734 nm. To perform the test, 0.15 mL of the sample extract was mixed with 2.85 mL of the prepared ABTS^•+^ solution. After incubation at 37 °C in darkness for 15 min, absorbance was measured at 734 nm using methanol as a blank.

#### 4.4.2. DPPH Assay

The antioxidant activity of the investigated extracts against DPPH (2,2-diphenyl-1-picrylhydrazyl) radicals was estimated as 0.15 mL of the extract, and 2.85 mL of a freshly prepared 0.1 mmol DPPH solution in methanol was added. After incubating the mixture at 37 °C in darkness for 15 min, absorption was measured at 517 nm, with methanol serving as the reference [19,20].

#### 4.4.3. CUPRAC Assay

The CUPRAC (Cupric Reducing Antioxidant Capacity) assay was conducted according the following procedure: the assay solution was a combination of 1 mL of a 10 mmol CuCl_2_ solution, 1 mL of 7.5 mmol neocuproine (Sigma) in methanol, 1.0 mL of 0.1 M ammonium acetate buffer (pH 7.0), 0.1 mL of the extract, and 1.0 mL of distilled water. The mixture was incubated at 50 °C in darkness for 20 min, and the absorption was measured at 450 nm against a reagent blank [19,21].

#### 4.4.4. FRAP Assay

The Ferric Reducing Antioxidant Power (FRAP) assay was carried out following the following method: the freshly prepared FRAP reagent was by mixing 10 parts of a 0.3 M acetate buffer (pH 3.6), 1 part of a 10 mmol solution of 2,4,6-tripyridyl-s-triazine (TPTZ, Fluka, Buchs, Switzerland) in 40 mmol HCl (Merck, Darmstadt, Germany), and 1 part of a 20 mmol FeCl_3_·6H_2_O solution (Merck) in distilled water. After that, the reaction was initiated by combining 3.0 mL of this reagent with 0.1 mL of the extract. The results were estimated after incubation at 37 °C in darkness for 10 min, and the absorption was measured at 593 nm against a blank sample from 70% ethanol [19,22].

### 4.5. Animals

This study was performed on 30 male Wistar rats weighing 200–220 g. The animals were obtained from the vivarium of the Medical University, Varna, Bulgaria. The animals were housed in stainless steel cages at 23 ± 2 °C in a well-ventilated room on a 12 h light/dark cycle. Rats had free access to standard rat chow and water. The humidity of the room was 50 ± 10%. All experimental procedures were performed between 8 and 10 a.m. The acclimatization of the animals was performed for one week. This study was carried out in accordance with the national requirements for the protection and humane treatment of laboratory animals, complying with Directive 2010/63/EU of the European Parliament and of the Council on the protection of animals used for scientific purposes. The performed experimental procedures were approved by the Bulgarian Food Safety Agency (Document № 175).

#### 4.5.1. Experimental Design

Rats were randomly divided into five groups of six animals each (n = 6). All chemicals, as well as EPE isolated from *Inonotus nidus-pici*, were administered orally by gavage. Indomethacin and famotidine were dissolved in 0.5% sodium carboxymethylcellulose (NaCMC), while the enriched fraction was dissolved in distilled water. Group 1 (control group) received 1 mL of saline orally for 14 consecutive days. Rats in group 2 received 1 mL of saline orally for 14 consecutive days and 1 mL of indomethacin (40 mg/kg) orally on the 15th day for induction of gastric ulcer (indomethacin group). Group 3 (positive control) received 1.25 mL of famotidine (20 mg/kg), while groups 4 and 5 received 1 mL of EPE solution at a dose of 25 mg/kg and 10 mg/kg, respectively, for 14 consecutive days prior to ulcer induction. Rats in groups 3, 4, and 5 also received 1 mL of indomethacin (40 mg/kg) orally on the 15th day. The animals were fasted for 24 h with free access to water prior to indomethacin administration. All rats were euthanized by cervical dislocation under diethyl ether anesthesia on the 15th day, four hours after indomethacin exposure. The stomach of each rat was immediately removed, opened along the great curvature, and washed in saline for macroscopic evaluation. Then, the stomachs were fixed in 10% neutral buffered formalin for histological analysis.

#### 4.5.2. Macroscopic Evaluation of Gastric Ulcers

Macroscopic assessment of gastric tissue was performed, and the number of gastric ulcers was determined. The severity of ulcer lesions was assessed through a score, as follows: 0 = no damage; 1 = blood at the lumen; 2 = pinpoint erosions; 3 = one to five small erosions < 2 mm; 4 = more than five small erosions < 2 mm; 5 = one or three large erosions > 2 mm; and 6 = more than three large erosions > 2 mm [23]. After calculating the score for each animal, the mean score for each group was determined. Therefore, the ulcer index (UI) for each group was calculated using the following equation [19]: UI = total ulcer score/number of animals. Additionally, the percentage of protection (PP) for each group of animals that were pretreated was calculated as follows [24]: PP = (UI of Indo group − UI of the pretreated group) × 100/UI of Indo group.

#### 4.5.3. Histopathological Evaluation of Gastric Mucosal Injury

The stomachs of all animals were fixed in 10% neutral buffered formalin, embedded in paraffin, separated into 4 µm thick slices using a rotary microtome, and stained with hematoxylin and eosin (H&E). A minimum of six fields from each stomach were examined. The severity of gastric injury was determined by an observer blinded to the animal treatment. All sections were evaluated for structural changes under a Leica DM 1000 LED light microscope and a Leica MC170 HD camera & SW Kit (Leica Microsystems AG, Wetzlar, Germany) and were captured at 200× or 400× magnification using the manufacturer’s software.

### 4.6. Statistical Analysis

All results are expressed as the arithmetic mean ± standard deviation (SD). The presented results are averages from two independent experiments carried out in triplicate. Statistical analysis was conducted with GraphPad Prism version 8.0.1 (GraphPad Software, San Diego, CA, USA). Data are presented as mean ± SEM. Differences between groups were analyzed using one-way analysis of variance (ANOVA) with Tukey’s multiple comparison post hoc test; Student’s t-test was also used. Values of *p* < 0.05 were considered statistically significant.

## 5. Conclusions

The quantification of main phenolic compounds and chromatographic analysis of *Inonotus nidus-pici* were conducted for the first time. Among all phenolic compounds, the best represented group was tannins as part of total polyphenols, comparable to that of phenolic acids. Ten phenolic acids were identified, with rosmarinic acid having the highest concentration. Flavonoids, like other phenols, were identified for the first time in the fungus and consist mainly of quercetin and (+)-catechin. The present study shows that the phenolic fraction, in addition to having antioxidant properties, could be used in the prevention of gastric ulcers when taken concomitantly with nonsteroidal anti-inflammatory drugs. More studies are needed to clarify the possible mechanism of the gastroprotective activity of different compounds in substances from this species.

The above-presented results can be a starting point for further chemical and pharmacological research on the substances of *Inonotus nidus-pici*.

## Figures and Tables

**Table 1:** HPLC analysis of main phenolics in EPE from *I. nidus-pici*.

Type of Phenols	Compounds	Content, mg/g DWE *
Phenolic acids	Syringic acid	0.70 ± 0.04 ^c^
	*p*-Coumaric acid	2.13 ± 0.12 ^b^
	Ferulic acid	1.70 ± 0.03 ^a^
	Salicylic acid	1.20 ± 0.02 ^b^
	Gallic acid	0.16 ± 0.09 ^c^
	Protocatechuic acid	0.86 ± 0.07 ^c^
	Chlorogenic acid	0.99 ± 0.06 ^a^
	Vanillic acid	0.81 ± 0.15 ^b^
	Caffeic acid	1.00 ± 0.16 ^b^
	Rosmarinic acid	6.41 ± 0.08 ^a^
Flavonoids	Rutin	0.44 ± 0.14 ^c^
	Hesperidin	0.83 ± 0.03 ^b^
	Kaempherol	1.67 ± 0.09 ^b^
	Quercetin	15.95 ± 0.05 ^a^
	(+)-Catechin	9.86 ± 0.15 ^b^
	(+)-Epicatechin	nd

* mg/g DWE—milligram per gram dry-weight extract; nd—not detected. Data are presented as mean ± standard deviation (SD). The results are from triplicate measurements. Values of the measured features with different small superscript letters are significantly different according to Tukey’s test (*p* < 0.01).

**Table 2:** Antioxidant capacity of EPE from *I. nidus-pici*.

ABTS (mM TE/g DWE)	DPPH (mM TE/g DWE)	FRAP (mM TE/g DWE)	CUPRAC (mM TE/g DWE)
723.15 ± 1.34	30.67 ± 0.25	1354.05 ± 2.87	1506.93 ± 2.61

**Table 3:** The ulcer index (UI) and the percentage of protection (PP) of different groups.

	Ulcer Index (UI)	Percentage of Protection (PP), %
Group I (control group)	0	-
Group II (IND)	4.67 ± 0.45	-
Group III (FAM + IND)	2.17 ± 0.61 **	53.57
Group IV (EPE, 25 mg/kg + IND)	2.83 ± 0.60 *	39.29
Group V (EPE, 10 mg/kg + IND)	4.17 ± 0.37	10.71

Data are presented as mean ± S.E.M. (n = 6). * *p* < 0.05 and ** *p* < 0.005 compared to the indomethacin group (Group II). S.E.M.—Standard error mean.

## Data Availability

The original contributions presented in this study are included in the article. Further inquiries can be directed to the corresponding author.

## Abbreviations

The following abbreviations are used in this manuscript:

HPLC	High-performance liquid chromatography
EPE	Enriched phenolic extract
DWE	Dry-weight extract
TE	Trolox equivalent
ABTS	2,2′-azino-bis(3-ethylbenzothiazoline-6-sulfonic acid
DPPH	2,2-diphenyl-1-picrylhydrazyl
UI	Ulcer index
FRAP	Ferric Reducing Antioxidant Power
CUPRAC	Cupric Reducing Antioxidant Capacity
